# Association of Serum Myeloid Cells of Soluble Triggering Receptor-1 Level with Myocardial Dysfunction in Patients with Severe Sepsis

**DOI:** 10.1155/2013/819246

**Published:** 2013-06-18

**Authors:** Fei Tao, Liangshan Peng, Juan Li, Yiming Shao, Liehua Deng, Huaguo Yao

**Affiliations:** Department of Critical Care Medicine, Affiliated Hospital of Guangdong Medical College, No. 57 Southern Renmin Avenue, Zhanjiang, Guangdong 524023, China

## Abstract

*Objective*. To investigate the association of serum sTREM-1 with myocardial dysfunction in patients with severe sepsis. *Methods*. A total of 85 patients with severe sepsis were divided into severe sepsis group (*n* = 40) and septic shock group (*n* = 45). Serum levels of sTREM-1, NT-proBNP, APACHE II score, SOFA score, cardiac index, cardiac function index, global ejection fraction, and left ventricular contractility index were measured on days 1, 3, and 7 after admission to ICU. *Results*. Serum sTREM-1 levels of patients with septic shock were significantly higher than those with severe sepsis on days 1, 3, and 7. Serum sTREM-1 was positively correlated with APACHE II scores, SOFA scores, and NT-proBNP. However, The sTREM-1 level was markedly negatively correlated with CI, CFI, GEF, and *dP*/*dt* max, respectively. Multiple logistic regression analysis showed that sTREM-1 was independent risk factor to NT-proBNP increasing. The optimal cut-off point of sTREM-1 for detecting patients with myocardial dysfunction was 468.05 ng/mL with sensitivity (80.6%) and specificity (75.7%). There is no difference in TREM-1-mRNA expression between the two groups. *Conclusions*. Serum sTREM-1 is significantly associated with myocardial dysfunction and may be a valuable tool for determining the presence of myocardial dysfunction in patients with severe sepsis.

## 1. Introduction

 Severe sepsis and septic shock are life-threatening complications of infections and the most common cause of death in intensive care units. About 50% of patients with sepsis show myocardial involvement characterized by biventricular enlargement, reduced contractility, and diastolic dysfunction [1]. This increases the risk of death and leads to an extremely poor prognosis in the case of severe sepsis or septic shock. Heart failure in severe sepsis or septic shock is currently regarded—although not yet classified—as a symptom of a secondary cardiomyopathy, which is characterized by an altered cellular phenotype due to the impact of multiple mediators and toxins [2].

Triggering receptor expressed on myeloid cells-1 (TREM-1) is a membrane molecule that is expressed on the surface of neutrophils and monocytes when they are triggered by bacteria and fungi. TREM-1 mediates the acute inflammatory response to microbial products. Human tissues infected with bacteria are infiltrate with neutrophils and monocytes that expressed high levels of TREM-1 [3, 4]. A soluble form of TREM-1 (sTREM-1) is released from the activated phagocytes and can be found in body fluids. Engagement of TREM-1 has been shown to induce the production of proinflammatory chemokines such as interleukin (IL-8) and cytokines such as tumour necrosis factor-*α* (TNF-*α*) and interleukin-1*β* (IL-1*β*) [5, 6], which have been implicated in ventricular dysfunction (depress ventricular contractility) associated with a variety of pathological conditions [7], including reperfusion injury and sepsis.

The N-terminal prohormone of brain natriuretic peptide (NT-proBNP) is a 76 amino acid N-terminal fragment of brain natriuretic peptide. NT-proBNP levels in the blood are used for screening, diagnosis of acute congestive heart failure (CHF) and may be useful to establish prognosis in heart failure, as the markers are typically higher in patients with worse outcome [8]. The serum concentrations of NT-proBNP are also typically increased in patients with asymptomatic or symptomatic left ventricular dysfunction and are associated with coronary artery disease and myocardial ischemia [9–11].

The purpose of this study was to track dynamic changes in serum sTREM-1, NT-proBNP levels and cardiac function in patients with severe sepsis or septic shock and to investigate the association of Serum myeloid cells of sTREM-1 levels with myocardial dysfunction in patients with severe sepsis.

## 2. Subjects and Methods

### 2.1. Subjects

After the study was approved by the local Ethics Committee, informed written consent was obtained from all subjects or a close kin of the patient. A total of 40 patients with severe sepsis and 45 patients with septic shock were included in the study taking place from May 2011 to November 2011. All patients were hospitalized in the Department of Intensive Care, Affiliated Hospital of Guangdong Medical College, and the required PiCCO monitoring was evaluated for inclusion. These patients were diagnosed with severe sepsis or septic shock according to the 1991 ACCP/SCCM Joint Meeting and by the diagnostic criteria developed at the 2001 International Sepsis Definition Conference.

Inclusion criteria were as follows:

-  the concomitant presence of age of 18 years and above,
-  severe sepsis or septic shock presented within the last 24 hours.

Exclusion criteria were as follows:

- neutropenia (≤500 neutrophils/mm^3^),
- patients with acquired immune deficiency syndrome,
- administration of corticosteroids defined as any oral intake of more than or equal to 1 mg/kg equivalent prednisone within the last month,
- a history of hypertension, coronary atherosclerotic heart disease, chronic heart failure, chronic renal insufficiency, or liver disease,
- recent history (7 days) of the following caused by elevated cardiac markers reasons: cardiothoracic surgery, external cardiac massage, defibrillation, and DC cardioversion.

Severe sepsis was determined as the acute dysfunction of at least one organ, that is, the acute presentation of at least one of the following:

- acute respiratory distress syndrome (ARDS), as any value of pO_2_/FiO_2_ below 200 with the presence of diffuse shadows in lung X-ray;
- acute renal failure, as the production of <0.5 ml/kg body weight/h of urine for at least two hours provided that the negative fluid balance of the patient was corrected;
- metabolic acidosis as any pH <7.30 or any base deficit greater than 5 mEq/L and serum lactate at least more than 2x normal value;
- acute coagulopathy as any platelet count <100 × 10^9^/l or INR > 1.5.

Septic shock was defined as systolic blood pressure below 90 mmHg for 1 hour requiring the administration of vasopressors provided that the negative fluid balance of the patient was corrected.

The patients were bundle treated referring to “the adult serious infection and septic shock hemodynamic monitoring and support guide” (2006).

The patients were divided into myocardial dysfunction group (NT-proBNP ≥ 450 pg/mL, age ≤50 years; NT-proBNP ≥ 900 pg/mL, age 50–75 years; NT-proBNP ≥ 1800 pg/mL, age ≥75 years) and nonmyocardial dysfunction group (NT-proBNP ≤ 450 pg/mL, age ≤ 50 years; NT-proBNP ≤ 900 pg/mL, age 50–75 years; NT-proBNP ≤ 1800 pg/mL, age ≥75 years) according to levels of NT-proBNP after 24 hours of admission to ICU.

### 2.2. Data Collection

Demographic and disease data of patients included age, gender, chief complaint for admission, vital signs, routine blood test results, liver and kidney functions, coagulation indicators, acute physiologic assessment and chronic health evaluation (APACHE) II scores, and sequential organ failure assessment (SOFA) scores. They were recorded on days 1, 3, and 7. Serum was collected at these same time points and sTREM-1 and NT-proBNP levels were determined.

We used the PiCCO system (PULSION medical system, Germany) for the hemodynamic monitoring. This system is a transpulmonary thermodilution method that can provide information on volume status, including cardiac index (CI, 3.0–5.0 L/min/m^2^), global ejection fraction (GEF, 25–35%), cardiac function index (CFI, 4.5–6.5 L/min), and left ventricular contractility index (*dP*/*dt* max,1200–2000 mmHg/s). These indicators can accurately reflect myocardial contractile function. A central venous catheter was inserted into the internal jugular vein or subclavian vein, and PiCCO arterial catheter was inserted into the femoral artery. Then, 10–15 mL normal saline at the temperature of <8°C was injected into the central vein, and various hemodynamic parameters can be obtained through analysis of variations in blood temperature taken by the temperature sensor of the arterial catheter. The recordings of hemodynamic parameters were carried out at least every 8 hours. After the first measurement, fluid management and the use of vasoactive agents were instituted according to the protocol of our institution. The first 8 hours was used as the study period and blood sampling for NT-proBNP was taken simultaneously at the first two transpulmonary thermodilution measurements.

### 2.3. Assays

Blood samples from patients were drawn from venous line for culture, and measurement of sTREM-1, NT-proBNP. After centrifugation, plasma was kept at −80°C until assayed. sTREM-1 was determined using a double antibody sandwich ELISA (Quan tikine Human TREM-1 Immunoassay ELISA Kit, R&D Systems, Minneapolis, MN, USA, product No. DTRM10B). NT-proBNP was measured by isotope label method.

A 3 mL quantity of peripheral whole blood was drawn from each subject on the first day. RNA was extracted with the selective binding properties of a silica-based membrane with the speed of microspin technology (Blood/Liquid Sample Total RNA Rapid Extraction Kit, Aidlab Biotechnologies). RNA was recognized after 3% agarose gel electrophoresis and ethidium bromide staining; 1.0 *μ*g of RNA was applied for the production of cDNA using PrimeScript RT reagent Kit with gDNA Eraser (Perfect Real Time) (Takara Biotechnology). cDNA was kept at −20°C until assayed.

Expression of mRNA was assessed by the LightCycler480 (Roche) using SYBR *Premix Ex Taq* II (Tli RNaseH Plus) (Takara Biotechnology). Primer sequences were the following: for TREM-1, sense 5′-GCT GTG GAT GCT CTT TGT CTC-3′ and antisense 5′-CAC TTG GAC TGG ATG GGA AT-3′, and for *β*_2_-microglobulin, sense 5′-ATG AGT ATG CCT GCC GTG TG-3′ and antisense 5′-CCA AAT GCG GCA TCT TCA AAC-3′. Amplification was followed by a melting curve; appropriate blanks were applied.

### 2.4. Statistical Analysis

Quantitative data with normal distributions, including age, APACHE II scores, and body temperature, are given as means ± standard deviations (SD). Student's *t*-test was used to compare means between two groups. Multiple logistic regression analysis was used to model the effect of the related sTREM-1 level, APACHE II scores, and SOFA scores on NT-proBNP level in patients with severe sepsis. Spearman correlation coefficients were used to assess associations between sTREM-1 levels and SOFA scores, and APACHE II scores, NT-proBNP. Receiver operating characteristic (ROC) curves for sTREM-1 levels for the myocardial dysfunction group and nonmyocardial dysfunction group were constructed based on statistically significant differences for calculating the areas under the ROC curves. Statistical analysis used SPSS Statistics 19.0. Any value of *P* below 0.05 after adjustment for multiple comparisons was considered statistically significant.

## 3. Results

### 3.1. Demographic Characteristics of Enrolled Patients according to the Clinical Stages of the Septic Syndrome

 Patients' ages, gender, and underlying diseases were not significantly different between the two groups (*P* > 0.05). However, the APACHE II scores and SOFA scores in the septic shock group were higher than those in severe sepsis group (*P* = 0.003 and *P* = 0.000, resp.), but the SBP and DBP in septic shock group were markedly lower than that in severe sepsis group, shown in Table 1.

### 3.2. Serum Concentrations of sTREM-1, NT-proBNP and CI, CFI, GEF, and *dP*/*dt* Max in Patients with Severe Sepsis and Septic Shock

Serum concentrations of sTREM-1 and NT-proBNP in the septic shock group were significantly higher than those in the severe sepsis group on days 1, 3, and 7. However, the CI, CFI, GEF, and *dP*/*dt* max in septic shock group were significantly lower than those in severe sepsis group on days 1, 3, and 7 (*P* < 0.05), shown in Table 2.

### 3.3. The Correlation of sTREM-1 Levels with APACHE II Scores, SOFA Scores, NT-proBNP, CI, CFI, GEF, and *dP*/*dt* Max

sTREM-1 levels were significantly positively correlated with APACHE II scores, SOFA scores, and NT-proBNP (*r* = 0.619, *P* < 0.05; *r* = 0.610, *P* < 0.05; *r* = 0.715, *P* < 0.05), respectively. However, sTREM-1 level was markedly negatively correlated with CI, CFI, GEF, and *dP*/*dt* max (*r* = −0.732, *P* < 0.05; *r* = −0.698, *P* < 0.05; *r* = −0.726, *P* < 0.05; *r* = −0.768, *P* < 0.05), respectively.

### 3.4. Multiple Logistic Regression Analysis

sTREM-1, APACHE II score, and SOFA score as independent variables and NT-proBNP as dependent variable, Multiple logistic regression analysis showed that serum sTREM-1 level in patients with severe sepsis was an independent risk factors to myocardial dysfunction (*r* = 0.619, 95%  CI:  0.842–1.550, *P* < 0.001), in Table 3.

### 3.5. Serum sTREM-1 to Diagnose the Myocardial Dysfunction

**Figure 1:**
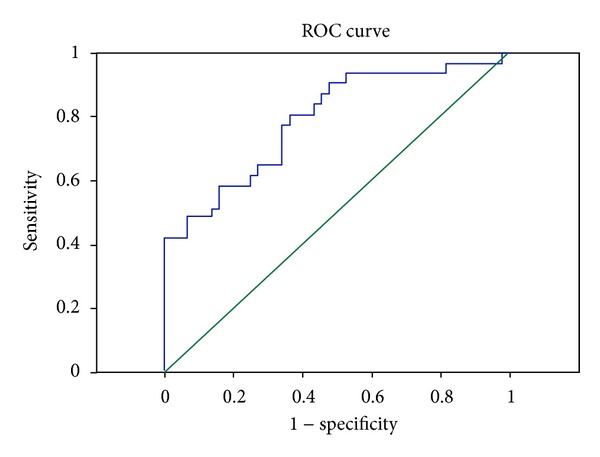
ROC curves of the sensitivity and specificity of sTREM-1 to discriminate between myocardial dysfunction and non-myocardial dysfunction in patients with severe sepsis.

The patients were divided into nonmyocardial dysfunction group and myocardial dysfunction group according to the levels of NT-proBNP after 24 hours of admission to ICU. ROC curves of the sensitivity and specificity of sTREM-1 to discriminate between myocardial dysfunction and nonmyocardial dysfunction in patients with severe sepsis are given in Figure 1. Areas under the receiver-operating characteristic curves were 0.79. 468.05 ng/mL as the cut-off point, the sensitivity, and specificity were 80.6% and 75.7%, respectively.

### 3.6. Gene Expression of TREM-1 among Patients with Severe Sepsis and Septic Shock

**Figure 2:**
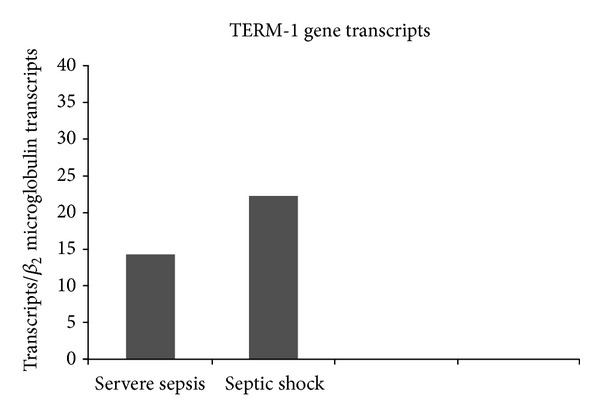
Gene transcripts of TREM-1 in Separated peripheral blood mononuclear cells (PBMCs) isolated from 40 patients with severe sepsis and from 45 patients with septic shock. *P* > 0.05 between severe sepsis group and septic shock group.

 Gene expression of TREM-1 among patients with severe sepsis and septic shock is shown in Figure 2. The number of TREM-1 gene transcripts was similar between severe sepsis and septic shock. No significant correlation was found between serum sTREM-1 and TREM-1 gene transcripts (*rs*: +0.196, *P*: nonsignificant).

## 4. Discussion

Sepsis and sepsis-induced mortalities are major health concerns worldwide. Septic shock is the most severe form of sepsis and is one of the most significant causes of death among critically ill patients. It is characterized by hemodynamic changes and the dysfunction of one or more organs. Cardiovascular changes are important in septic shock; peripheral vascular dysfunction, which can result in heterogeneous microcirculatory flow, can frequently induce myocardial depression. In this population, cardiovascular collapse can increase the risk of death in sepsis as much as two times, and myocardial depression occurs in almost 40% of septic patients. Myocardial depression is characterized by a cardiac output that fails to meet metabolic demands [12, 13].

Triggering receptor expressed on myeloid cells-1 (TREM-1), discovered by Bouchon et al. in 2000 [4], is a member of the immunoglobulin superfamily of receptors that is specifically expressed on the surfaces of monocytes and neutrophils. Its soluble form, named sTREM-1, has been detected in cell culture supernatants after stimulation of neutrophils and monocytes with endotoxins as well as in sera of patients with sepsis [14, 15]. The exact role of sTREM-1 in the pathogenesis of sepsis is yet undefined. The present study was focused on the differences of the levels of sTREM-1 in severe sepsis and septic shock patients and investigated the correlation of sTREM-1 with myocardial dysfunction.

In the present study, the serum concentrations of sTREM-1 in septic shock patients were significantly higher than those with severe sepsis on days 1, 3, and 7. Furthermore, a positive correlation was detected between sTREM-1 and the APACHE II scores or SOFA scores. The results demonstrate that sTREM-1 has a positive correlation with illness severity in patients with sepsis, and sTREM-1 might be involved in septic shock development.

The present study also showed that sTREM-1 level was markedly positively correlated with NT-proBNP and negatively correlated with CI, CFI, GEF, and *dP*/*dt* max. Multiple logistic regression analysis showed that serum sTREM-1 levels were independent risk factors to NT-proBNP increasing. Therefore, we hypothesized that sTREM-1 levels increased in serum might be involved in myocardial dysfunction in patients with severe sepsis or septic shock. There are several possible reasons for this observation. Firstly, TREM-1 induces secretion of tumor necrosis factor (TNF-*α*) and interleukin-1*α*, interleukin-6, which may result in cardiac impairment. Both microbial and endogenous proinflammatory mediators, such as bacterial endotoxin (lipopolysaccharide (LPS)), IL-1, and TNF-*α*, may directly depress myocardial contraction [16]. It was found from the cytological experiments that proinflammatory cytokines have the effects of stimulating NT-proBNP synthesized on cardiomyocytes [17]. Several candidates with a potential pathogenetic impact on the heart were identified: bacterial toxins; cytokines and mediators; nitric oxide; cardiodepressant factors; oxygen reactive species; and catecholamines.

Our study found that CI, CFI, GEF, and *dP*/*dt* max in septic shock group were significantly lower than those in severe sepsis group on days 1, 3, and 7, but regardless of severe sepsis group and septic shock group, these index was slightly decreased. Those results are consistent with those reported by Zhou et al. [18], who reported that cardiac contractility and structure are not significantly compromised even during the late, hypodynamic stage of sepsis. There are several possible reasons for this observation. First, cardiac function is depressed during endotoxic shock; however, septic shock in humans is usually characterized by a high cardiac output, a low systemic vascular resistance, and a hyperdynamic hemodynamic profile [19–21], so, ventricular contractility is not altered at early phase of sepsis. Besides, during human septic shock, reversible depression of left ventricular ejection fraction and dilatation of the left ventricle have been described using radionuclide angiography or echocardiography. These changes in left ventricular function and size are transient and return toward normal in survivors at seven to ten days after the onset of septic shock. In addition, sample size too little in our research, which may influence the result.

The patients were divided into nonmyocardial dysfunction group and myocardial dysfunction group according to the levels of NT-proBNP after 24 hours of admission to ICU. We further evaluated the value of sTREM-1 for diagnosing whether or not patients are with myocardial dysfunction. Areas under the receiver-operating characteristic curves were 0.79. 468.05 ng/mL as the cut-off point, the sensitivity, and specificity were 80.6% and 63.6%, respectively. The low sensitivity value may have been related to the small sample size, and follow- up studies may include larger samples.

The present study also describes the level of gene expression of TREM-1 in monocytes. The number of TREM-1 gene transcripts was similar between severe sepsis and septic shock. Our results are consistent with those reported by Dimopoulou et al. [22], who reported that gene expression of TREM-1 on monocytes in severe sepsis/shock is not increased compared with patients with sepsis. Serum sTREM-1 increases early in sepsis at levels related to disease severity, which is not the case for TREM-1 gene transcripts. This is consistent with the lack of statistical correlation between sTREM-1 in serum and TREM-1 gene transcripts of monocytes. The origin of sTREM-1 is so far unclear; two hypotheses have been advanced to explain the origin of sTREM-1: alternative splicing of TREM-1 mRNA and proteolytic cleavage(s) of mature, membrane-anchored TREM-1. Gómez-Piña et al. [23] found that proteolytic cleavage of membrane-anchored TREM-1 by one or several matrix metalloproteinases is responsible for sTREM-1 generation. But our finding highlights that circulating sTREM-1 is affected by factors more complex than single gene transcription of TREM-1 like the rate of shedding of the membrane-bound receptor.

## 5. Conclusions

In summary, serum sTREM-1 levels correlated with disease severity and independent risk factors to myocardial dysfunction; it may be as a valuable tool for determining the presence of myocardial dysfunction in patients with severe sepsis. These findings add considerably to our knowledge on the pathophysiological response of the severe sepsis patients with myocardial dysfunction.

## Figures and Tables

**Table 1:** Demographic characteristics of patients with severe sepsis and septic shock.

	Severe sepsis	Septic shock	*P* value
	(*n* = 40)	(*n* = 45)	
Age	63.1 ± 16.7	65.3 ± 16.8	0.547
SBP (mmHg)	115.8 ± 32.7	82.3 ± 15.4	0.000
DBP (mmHg)	62.6 ± 8.7	52.3 ± 6.4	0.000
APACHE II scores	14.08 ± 5.55	17.44 ± 8.51	0.003
SOFA scores	6.04 ± 2.45	9.12 ± 3.68	0.000
Infections (*n*, %)			
Lung	24 (60%)	28 (64%)	
Abdomen	10 (25%)	11 (24%)	
Others	6 (15%)	6 (12%)	

SBP: systolic blood pressure; DBP: diastolic blood pressure; APACHE II scores: acute physiology and chronic health evaluation II score; SOFA scores: sequential organ failure assessment score.

**Table 2:** Serum concentrations of sTREM-1, NT-proBNP, and CI, CFI, GEF, and *dP*/*dt* max in patients with severe sepsis and septic shock on days 1, 3, and 7.

Group	Severe sepsis (*n* = 40)			Septic shock (*n* = 45)		
	1 day	3 days	7 days	1 day	3 days	7 days
sTREM-1 (pg/mL)	473.14 ± 260.84	392.11 ± 154.05	262.58 ± 133.32	1070.33 ± 545.61^#^	928.97 ± 638.53^*※*^	693.62 ± 485.87^▲^
NT-proBNP (pg/mL)	595.28 ± 372.80	428.84 ± 363.34	245.28 ± 195.20	1743.32 ± 1230.9^#^	1218.78 ± 1125.30^*※*^	963.30 ± 1029.61^▲^
CI	3.00 ± 0.72	3.35 ± 0.81	3.65 ± 0.78	2.62 ± 0.78^#^	2.78 ± 0.98^*※*^	3.13 ± 1.21^▲^
CFI	4.15 ± 1.37	4.63 ± 0.97	5.10 ± 1.22	3.45 ± 1.30^#^	4.10 ± 1.14^*※*^	4.76 ± 0.78
GEF	15.80 ± 6.25	18.4 ± 6.59	21.50 ± 8.47	13.20 ± 4.80^#^	15.93 ± 4.10^*※*^	22.87 ± 6.64
*dP*/*dt* max	1219.50 ± 484.91	1413.90 ± 335.47	1781.50 ± 463.43	993.95 ± 414.86^#^	1194.60 ± 433.16^*※*^	1505.07 ± 464.29^▲^

NT-proBNP: N-terminal probrain natriuretic peptide; CI: cardiac index; CFI: cardiac function index; GEF: global ejection fraction; *dP*/*dt* max: left ventricular contractility index; ^#^ *P* < 0.05 compared with severe sepsis group on day 1, ^*※*^ *P* < 0.05 compared with severe sepsis group on day 3, ^▲^ *P* < 0.05 compared with severe sepsis group on day 7.

**Table 3:** Multiple logistic regression analysis.

Variable	*b* value	*t* value	*P* value
sTREM-1	0.619	6.739	0.000
APACHE II scores	0.094	0.915	0.363
SOFA scores	0.116	1.025	0.309

sTREM-1, APACHE II, and SOFA score as independent variables and NT-proBNP as dependent variable.
